# The Lords' Committee

**Published:** 1891-07-11

**Authors:** 


### The Lords' Committee.

On Saturday last a special sitting was held, when Mr. Henry C. Burdett gave evidence. Mr. Burdett, who was examined by the Chairman, said he was entirely opposed to rate-supported hospitals as distinguished from voluntary hospitals. There had been a great cry latterly that the only thing we could do for our hospitals was to make them rate-supported. He was thoroughly of opinion that the day when England did make them all rate-supported would be the worst day that the sick people of England ever saw. He went on to say that the interest in hospitals was of comparatively recent growth, and when he first entered a hospital they were more like workhouses than hospitals. By Lord Kimberley: It was unnecessary to have outside inspection, although he admitted that inspection was of value in the case of the existing hospitals. Lord Kimberley: If inspection is of value in hospitals, why is it your opinion that hospitals should go on without a regular system of inspection? Witness: Because public opinion is sufficient to keep them efficient. Although he considered that rate-supported hospitals were not well managed, he would say that the Poor-law infirmaries were of two distinct classes, some excellently administered, and some not so well administered. At the Marylebone Infirmary, the Paddington Infirmary, and many others which were instituted under Hardy's Act, everything was as complete and efficient and excellent as could be found at any hospital in the world; they had a most splendid equipment which left little to be desired; and the officers were in every way as efficient as they could be. He had lately visited the new Poor-law infirmary in Birmingham, which was remarkable in many ways, especially with reference to its nursing work. He would advise the Committee to take an opportunity of making a personal visit to that institution. He had tried to obtain plans of the beds at the Hospital for Incurables at Putney, and had been refused at every turn; even in Russia he had never met with such difficulties on trying to enter a hospital. He would propose that a voluntary body such as the Hospital Sunday Fund Council might best undertake, with the aid of the Council and a Visitor, the inspection of hospitals. With reference to annual subscriptions being such a small portion of the income of many hospitals, Mr. Burdett said he thought that was due to the authorities not understanding the power of the guinea. At the Seamen's Hospital the income had risen £6,000 in five years, because the secretary had looked up the guinea subscribers. Mr. Burdett handed in some extraordinary tables, showing the percentage of in and out patients to the population of the various towns of the kingdom. Also one giving the number of hospital beds to patients in the chief cities of the world. A large map of London showing the position of the hospitals was also exhibited, and Mr. Burdett explained that the distribution of the hospitals was all wrong. Within the two-mile radius from Charing Cross were three-quarters of all the beds of the London hospitals. What ought to be done was to have outpost hospitals all over London such as the Seamen's had started, where urgent accidents could be admitted, while less serious cases could be passed on to the parent institution, which should be connected by telephone, and ambulance service. Other plans put in showed how the sites of such hospitals as University College and Charing Cross did not include the full square, as should be done; a philanthropist could not do better than present these institutions with the remainder of their site. With regard to the out-patient departments, the witness thought it would be well if they could be closed altogether for a time, so greatly are they abused. There was an excellent system in America by which the burden of proof that he was a fit subject for medical charity was thrown on the patient. For teaching purposes the poor-law dispensaries would be as excellent as the out-patient department. All patients should be granted the privilege of being allowed to pay what they could; the home or pay hospital was an excellent institution, especially Fitzroy House. Lord Cathcart: I may mention that I have twice been to Fitzroy House, once for a gun-shot wound obtained in Egypt; I was admirably treated on both occasions. Asked to define who were the proper recipients of medical charity, witness said he thought only those who could keep themselves, save when sickness caused a great increase of expense. Lord Sandhurst: Now about your Pension Fund for Nurses? The best table is table F, which provides sick pay and pension on the returnable rate. We calculated that a nurse having no rent to pay, could afford to put by an eighth of her income; taking a nurse of 25 years of age, receiving £25 a-year, if she puts by £4 2s. 0d. she is eligible for 10s. a-week in sickness, and a pension of 10s. a-week after the age of sixty. The witness also gave particulars of the rise and extraordinarily rapid progress of the Fund. With regard to the proposed registration of nurses the witness said that practically a proper system of registration already existed, for the hospitals which trained the nurses gave them certificates when the nurses were efficient. The proposal that an outside body should undertake a system was unnecessary, and events had already shown, could only work to the detriment of good nurses. Mr. Burdett mentioned one case in which an unsatisfactory nurse was refused her certificate by her school, but was now going about backed up by the certificate of this outside body, and by the name of the Princess Christian.

---

Dr. V. N. Ergolski, writing in the *Vratch,* reports several cases of chronic alcoholism which have been treated with more or less success by means of strychnine. In one case a man on the fifth day of treatment attended the name-day festival of a relative, when he was urged to drink the health of the host. He felt such a loathing for drink that he refused. At the end of fifteen months he appears to have been perfectly cured.

